# Randomised controlled trial of an online mental health and suicide gatekeeper resource for parents and caregivers: study protocol

**DOI:** 10.1136/bmjopen-2023-082963

**Published:** 2024-07-17

**Authors:** Alison L Calear, Sonia M McCallum, Dominique Kazan, Michelle Torok, Aliza Werner-Seidler, Bridianne O'Dea, Alyssa Morse, Louise Farrer, Fiona Shand, Philip J Batterham

**Affiliations:** 1 Centre for Mental Health Research, The Australian National University, Canberra, Australian Capital Territory, Australia; 2 Black Dog Institute, University of New South Wales, Sydney, New South Wales, Australia

**Keywords:** MENTAL HEALTH, Adolescents, Caregivers, Child, Randomized Controlled Trial, Suicide & self-harm

## Abstract

**Introduction:**

Rates of help-seeking for mental disorders and suicide are low among children and adolescents. Parents are viewed as gatekeepers for their care, yet they may lack the knowledge and skills to identify needs or facilitate service access. The primary aim is to test the effect of a new gatekeeper resource for parents and caregivers on their self-efficacy to recognise, respond and access support for mental health problems and suicide risk in their child.

**Methods and analysis:**

A two-arm randomised controlled trial will compare an online mental health and suicide gatekeeper resource for parents and caregivers to a waitlist control. Australian parents of children aged 5–17 years recruited through social media and community advertising will participate in an online trial. Participants randomised to the intervention condition will be emailed the resource to work through at their own pace. The resource consists of three sections providing parents and caregivers with confidence, knowledge and skills to recognise and respond to mental health problems and suicide risk in their child, as well as support them in accessing professional help. The primary outcome measure is self-efficacy to recognise, respond and provide support for mental health problems and suicide risk, while secondary outcomes include perceived knowledge, stigma, literacy, help-seeking attitudes, intentions and barriers. Data will be collected at preintervention, postintervention (4 weeks after accessing the resource) and 12-week follow-up. Primary analyses will compare changes in self-efficacy in the intervention condition relative to the waitlist control using mixed-model repeated measures analyses.

**Ethics and dissemination:**

The ethical aspects of the study were approved by the Australian National University Human Research Ethics Committee (Protocol 2023/195). If effective, the resource will fill an important gap in resources for parents, with the potential for dissemination through school groups, community organisations and clinical settings.

**Trial registration number:**

Australian New Zealand Clinical Trials Registry, ACTRN12623000933651.

**STRENGTHS AND LIMITATIONS OF THIS STUDY**

- The mental health and suicide gatekeeper resource trialled in the current study has been developed in collaboration with parents and caregivers to ensure it meets their needs.
- The resource will be evaluated using a rigorous randomised controlled trial methodology.
- Quantitative and qualitative outcome measures are included in the study to enable statistical changes in outcomes to be assessed, as well as to provide participants with a voice with which to share their experiences using the intervention.
- The recruitment of fathers to parenting research can be challenging, so the study is unlikely to have an equal representation of male and female caregivers.
- While the current study will assess intervention effects up to 12 weeks postintervention, longer-term retention of knowledge and skills will not be assessed. It is not feasible to assess the impact on child mental health outcomes.

## Introduction

 Mental health problems, suicide and self-harm are significant public health concerns in children and adolescents. A recent meta-analysis of the global prevalence of mental disorders in children and adolescents found the prevalence of anxiety and depressive disorders to be 6.5% and 2.6%, respectively,^1^ while a comprehensive review of suicide phenomena in young people worldwide estimated the proportion of adolescents reporting lifetime suicidal thoughts or a suicide attempt at 29.9% and 9.7%, respectively.^2^ Timely identification of mental health problems, self-harm and suicide risk is vital, as earlier access to treatment and support is associated with improved outcomes and reduced distress.^3^

For many children and adolescents, their parents or primary caregiver are often the primary source of support in times of distress. Parents are also likely to be best placed to recognise changes in their child’s behaviour or identify when a problem exists, particularly among younger children.^4 5^ As such, parents can play a fundamental role in supporting the emotional well-being of their child and assisting them to seek and access help for mental health problems when they arise. Given that parents are important ‘gatekeepers’ to their child’s health and well-being, parental knowledge and awareness of mental health in children and adolescents, and their attitudes towards mental disorders and help-seeking, will influence whether care is sought.

There is great variability in parental knowledge about child mental health; some parents do not know when or how best to respond if their child experiences emotional distress, while for other parents, their own attitudes towards mental health problems or services may prevent them from acting.^6^ In a cross-sectional survey conducted with parents across Australia, high suicide stigma and low suicide literacy were associated with poorer help-seeking attitudes and lower intentions to seek professional help for a child expressing thoughts of suicide.^7^ Similar findings were found in a second study assessing the role of parental knowledge and attitudes in professional help-seeking for child anxiety.^8^ In this study, lower anxiety stigma was associated with help-seeking from a general practitioner (GP) or psychologist.^8^ While parents tend to be aware of help-seeking options for young people in distress, such as school counsellors or GPs,^9^ their confidence in facilitating referral to care or in recognising the signs of distress and suicide is typically low due to a lack of intervention programmes supporting parent knowledge in this area.^10 11^

Gatekeeper training interventions are designed to improve the recognition and referral of people at risk of suicide or psychological distress and have been found to improve confidence in supporting a person in distress, reduce stigma and increase the recognition and referral of individuals at risk of suicide or distress.^12 13^ Gatekeeper interventions have traditionally been delivered face-to-face and require several hours to complete, which may be barriers for time-poor parents.^14^ Therefore, there is a need to develop more accessible online gatekeeper interventions that are tailored specifically to the needs of parents, can be used flexibly at a time that works for parents and is evaluated using rigorous research methods.^12 13^ To date, few ‘brief’ gatekeeper training interventions have been purpose-designed for, or evaluated with, parents.^12 15^

Recently, a pre-post study was conducted to assess the efficacy of a generic online suicide prevention training programme in a sample of parents of young people aged 12–25 years.^16^ The results of this study showed increases in participant self-efficacy and formal help-seeking intentions, reductions in suicide stigma at postintervention and increases in suicide literacy at 3-month follow-up. While the results of this study are promising, the constrained focus on suicide, the use of a programme not explicitly tailored for parents, and the pre-post design of the study are inherent limitations.

In order to address these limitations, a new online gatekeeper intervention was developed in collaboration with end users, which specifically targets parents and caregivers and addresses common mental health problems in children and adolescents, as well as suicide and self-harm. To ensure its relevance to a wider range of parents and caregivers, the intervention was also designed to have a dual focus on upskilling parents and caregivers prior to difficulties arising through the building of knowledge and supportive attitudes, as well as teaching skills to respond to and support a young person with existing mental health problems.

### Aims and hypotheses

Using randomised controlled trial (RCT) methodology, this study will investigate the effectiveness and acceptability of the new resource for parents and caregivers of children aged 5–17 years. The primary aim of the trial is to assess the effect of the resource on participant self-efficacy to recognise, respond and access support for mental health problems and suicide risk in their child at postintervention (primary endpoint) and at 12-week follow-up, compared with participants in the waitlist control condition. It is hypothesised that participants in the intervention condition, who receive the resource, will report higher levels of self-efficacy at postintervention and follow-up than participants in the control condition.

The secondary aims of the trial include (1) evaluating differences at postintervention and 12-week follow-up between the intervention and waitlist control conditions on perceived gatekeeper knowledge, mental health and suicide literacy, mental health and suicide stigma, and help-seeking barriers, attitudes and intentions and (2) assessing user satisfaction and acceptability of the mental health and suicide gatekeeper resource. In relation to the former, it is hypothesised that at postintervention and 12-week follow-up, participants in the intervention condition will report higher levels of perceived gatekeeper knowledge, mental health and suicide literacy, help-seeking attitudes and intentions, and lower levels of mental health and suicide stigma and help-seeking barriers than participants in the waitlist control condition. Finally, it is hypothesised that a brief online resource will be an acceptable method of delivering a gatekeeper resource to this participant group.

## Methods and analysis

This study protocol complies with the Standard Protocol Items: Recommendations for Interventional Trials (SPIRIT) guidelines.

### Study design

A two-arm RCT with an intervention condition (mental health and suicide gatekeeper resource) and a waitlist control condition will be conducted. Outcome measures will be administered on three occasions: preintervention, postintervention (4 weeks after receiving access to the intervention) and 12-week follow-up.

### Recruitment

Participants eligible to participate in this study will be Australian residents aged 18 years and over who are a parent or caregiver of a child aged 5–17 years. Participants are required to have sufficient English literacy skills and access to a computer, smartphone or tablet in order to complete the training and surveys. Parents of children with and without mental health problems will be eligible to participate. Parents will be recruited to the trial through social media platforms, as well as through school newsletters, other community settings and an established research register of parents interested in mental health research (which is managed by the research team at the Australian National University). Within the social media platforms, participants will be recruited through paid advertisements and promotion of the trial among established parenting groups and the social media pages managed by the research team and their respective institutions. In all cases, participants will be provided with a link to an online portal that hosts information about the trial, assesses trial eligibility, collects digital consent and administers the preintervention survey. Participants will provide their email addresses to enable enrolment in the online resource and the administration of postintervention and follow-up surveys. Rolling recruitment of participants will occur until the required sample size is reached.

### Randomisation

After consenting to participate in the trial and completing the preintervention survey, each participant will be individually randomised to the intervention or waitlist control condition. Stratified randomisation will be conducted by an independent statistician who is not involved in the day-to-day management of the trial according to ICH guidelines,^17^ using a computerised random number generator with 1:1 allocation to the intervention and waitlist control conditions using predetermined block sizes of 6. Randomisation will be stratified by participant gender (female vs male, non-binary, use different terms, prefer not to answer) and child with the experience of a mental health problem (yes vs no) to ensure an even balance of mothers and fathers and pre-existing child mental health exposure across conditions.

### Procedure

After randomisation, all participants will be informed of their trial condition via the email address they provided in the preintervention survey. At this time, participants allocated to the intervention condition will be provided with instructions and login details to access the mental health and suicide gatekeeper resource via an online learning platform. Participants will be provided with access to the resource over a 4-week period and encouraged to work through it in several sittings. For intervention-condition participants who have not completed the resource, weekly email reminders to access the resource will be sent.

During the intervention phase, the trial participants in the waitlist control condition will continue their usual activities. After the 4-week intervention period, all participants will be invited to complete an online postintervention self-report survey. Participants will be invited to complete a third and final online self-report survey at 12 weeks postintervention. Participants will receive notification of the postintervention and follow-up surveys via email and will receive up to two email reminders if the surveys are not completed. Surveys will take approximately 20–30 min to complete. To assist with participant retention in the trial, all participants will be offered a AU$15 egift card for completing the postintervention and follow-up surveys, which will be sent to participants via the email details provided during registration. Upon completion of the trial period, participants in the waitlist control condition will be provided access to the mental health and suicide gatekeeper resource and will have access to it for 4 weeks.

**Figure 1:**
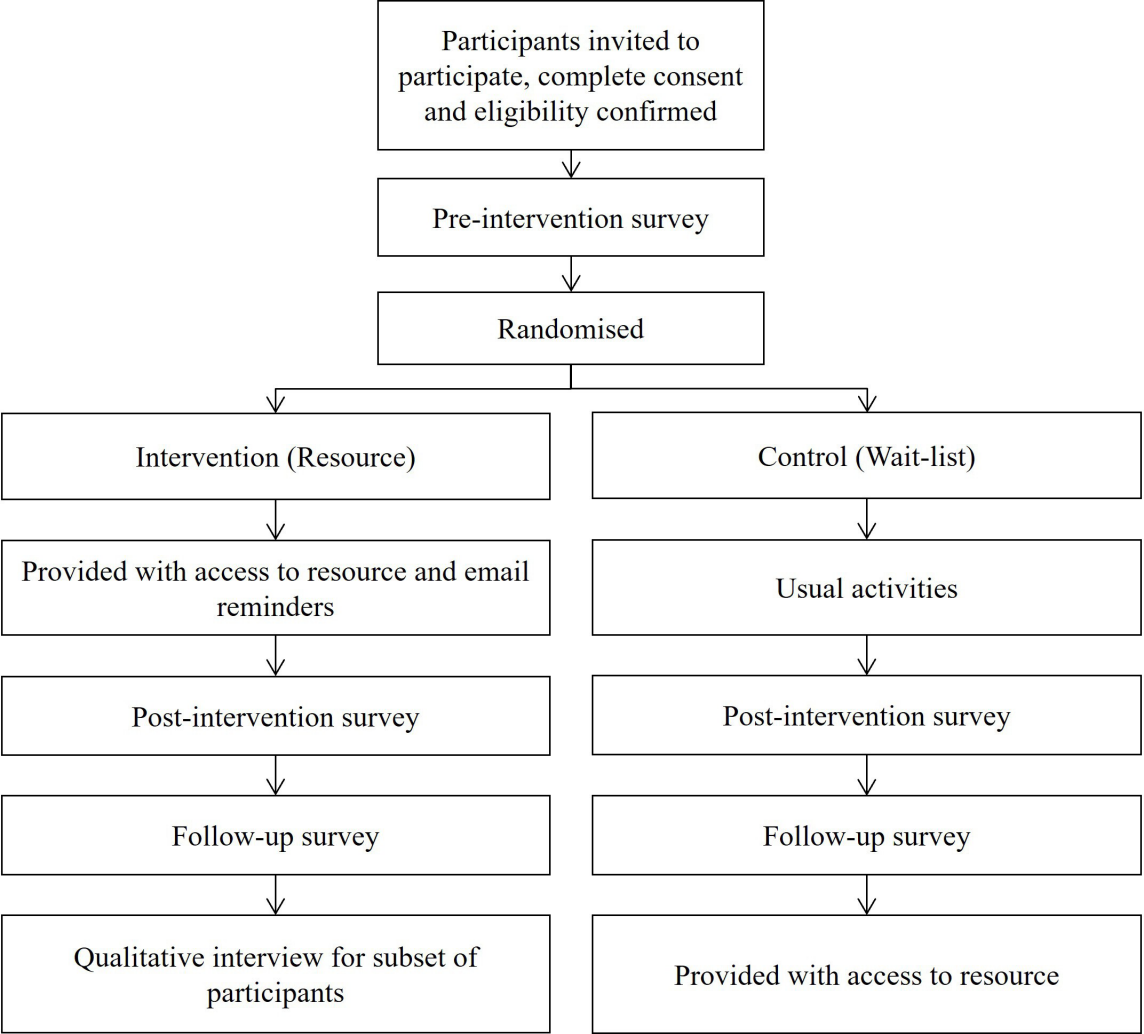
Participant flow in the trial.

A subset of participants who were allocated to the intervention condition will also be invited to participate in a qualitative interview. Participants will have the opportunity to indicate their interest in undertaking an interview at the end of the follow-up survey. The online interview will take up to 60 min to complete and will consist of a number of open-ended questions to stimulate discussion on parent views of the resource, guide future refinement of the resource and identify optimal dissemination pathways. Participants will be offered a AU$40 egift card to compensate them for their time. The flow of participants within the trial is presented in figure 1.

### Intervention

*Recognise,Respond andSupport—AParent’sGuide toYouthMentalHealth* is a newly developed digital mental health and suicide gatekeeper resource that was designed to assist parents and caregivers in supporting their child’s mental health. This interactive digital resource is targeted to parents and caregivers of young people aged 5–17 years and provides evidence-based information, lived experience perspectives and help-seeking guidance and resources. Taking between 90 and 120 min to complete in full, the resource consists of three key components: *Recognise*, *Respond* and *Support*. To meet the individual preferences of participants, the resource can be completed in one or multiple sittings. However, participants will be encouraged to complete the resource over multiple sittings to facilitate learning and reflection, with opportunities to take a break flagged in the resource.

The *Recognise* section aims to provide parents and caregivers with the knowledge to recognise mental health problems or suicide risk in their child and assist parents in identifying when external professional help may be needed. This component of the resource begins with a brief introduction to mental health, the ‘well to unwell’ mental health continuum and potential risk factors for poor mental health. Specific information is then provided on anxiety, depression, self-harm and suicide, as well as how to identify when help may be needed. Separate signs of anxiety and depression are listed for children and adolescents to provide age-specific information for parents.

The next component of the resource, *Respond*, is designed to provide parents and caregivers with guidance on what to do if they have concerns about their child’s mental health and how to respond to a disclosure of suicide or psychological distress. This section provides information and strategies on preparing for and initiating a conversation with their child, including tips from young people, what to do if the young person does not want to talk, specific guidance on asking about and responding to disclosures of self-harm and suicide and safety planning.

The *Support* component of the resource aims to provide parents with information and step-by-step guidance on accessing professional help and support for their child’s mental health and what to do in a crisis situation. This section of the resource begins with an acknowledgement of common reactions parents and caregivers may experience upon learning of mental health difficulties in their child. Information on key mental health professionals is provided, as well as guidance on accessing support from the child’s school and strategies to create a supportive home environment that facilitates open discussions about mental health. The importance of looking after oneself while managing a child’s mental health difficulties is also emphasised. Downloadable information sheets are available throughout the resource and direct links to these are provided at the conclusion of the resource (see online supplemental material for resource screenshots).

### Patient and public involvement

The *Recognise, Respond and Support— A Parent’s Guide to Youth Mental Health* resource was developed in response to previous research conducted with parents and caregivers in which the need for further information and guidance on supporting child mental health difficulties and suicide risk was identified.^10^ This led to the recruitment of a diverse sample of parents and caregivers through social media to engage in the completion of a survey (n=631) to identify the target disorders (anxiety, depression, self-harm and suicide) and population (both children and adolescents) of the resource, as well as its format (digital self-help), key informational needs in the areas of recognition, response and support, and an assessment of potential target areas (literacy and stigma) to identify areas for improvement. A series of interviews (n=14) were then conducted with parents and caregivers of young people who had a history of mental health problems to understand their help-seeking journey and what information would have been helpful for them at the time to better navigate the process. Based on the information gathered during these engagement activities, as well as other research conducted with parents and caregivers^7 8^ a digital resource was developed. This resource was then reviewed by parents and caregivers (n=9) and changes were made in response to their feedback, leading to the final resource now being evaluated.

### Survey measures

The administration schedule for each of the survey measures and the primary and secondary outcomes are presented in table 1.

**Table 1:** Survey measures and timing

Measure	Preintervention	Postintervention	12-week follow-up
Demographics	✓		
Child mental health	✓		✓
Primary outcome			
Self-efficacy	✓	✓	✓
Secondary outcomes			
Perceived gatekeeper knowledge	✓	✓	✓
Mental health literacy	✓	✓	✓
Anxiety literacy			
Depression literacy			
Suicide literacy	✓	✓	✓
Knowledge of depression and suicide	✓	✓	✓
Mental illness stigma	✓	✓	✓
Suicide stigma	✓	✓	✓
Help-seeking attitudes	✓	✓	✓
Barriers to help-seeking	✓	✓	✓
Help-seeking intentions	✓	✓	✓
Psychological distress	✓	✓	✓
Resource satisfaction, acceptability and engagement		✓	✓

#### Self-efficacy

The primary outcome measure in the current trial is self-efficacy in being able to recognise, respond and access support for mental health problems and suicide risk at postintervention. Ten items were adapted from the Parent Self-Efficacy Scale^11^ and Confidence in Helping Scale^18^ to reflect the objectives of the resource, with five items assessing mental health self-efficacy and five assessing self-harm and suicide self-efficacy. Within each set of items, participants are asked to rate their level of confidence in recognising signs and symptoms, initiating conversations, responding to disclosures, providing support to their child and seeking professional help. Items are rated on an 11-point scale ranging from 0 (not at all confident) to 10 (completely confident), with a midpoint of 5 (somewhat confident). Higher scores indicate greater levels of self-efficacy.

#### Perceived gatekeeper knowledge

The level of perceived gatekeeper knowledge is assessed using a 15-item bespoke measure that was based on the self-evaluation of knowledge questions used in the *Question, Persuade, Refer* gatekeeper training assessments.^19^ Items were adapted for suitability in parents reporting on knowledge for supporting their children. Eleven items assess perceived gatekeeper knowledge associated with recognising, responding and supporting mental health problems in children and adolescents (eg, signs and symptoms of mental health problems in children), while the remaining four items assess perceived gatekeeper knowledge associated with self-harm and suicide (eg, how to respond to self-harm). Items are rated on an 11-point scale ranging from 0 (very low) to 10 (very high), with higher scores indicating greater levels of perceived gatekeeper knowledge.

#### Mental health literacy

Four items from the Anxiety Literacy Questionnaire^20^ and four items from the Depression Literacy Questionnaire^21^ will be used to assess participant mental health literacy, with a particular focus on the recognition of the signs and symptoms of anxiety and depression. Each item is responded to on a 3-point scale (true, false and don’t know), with the correct responses allocated one point. Total scores on each scale range from 0 to 4, with higher total scores indicating greater knowledge of anxiety or depression.

#### Suicide literacy

Parents’ knowledge of suicide literacy will be assessed with four items from the Literacy of Suicide Scale,^22^ with response options of true, false or don’t know. Correct responses are allocated a score of 1, with total scale scores calculated by summing item scores. Total scale scores range from 0 to 4, with higher scores indicating greater knowledge of suicide prevention.

#### Knowledge of depression and suicide

To assess specific knowledge of child and adolescent mental health, the 7-item Parent Knowledge of Depression and Suicide Scale,^23^ will be administered. Items on this measure are rated on a 4-point scale ranging from ‘strongly disagree’ to ‘strongly agree’, with a fifth ‘don’t know’ response option. Correct responses (agreement or disagreement, depending on the item) are allocated a score of one. Incorrect and ‘don’t know’ responses are allocated a score of 0. Individual item scores are summed to create a total scale score between 0 and 7, with higher scores reflecting greater knowledge.

#### Mental illness stigma

Negative attitudes towards people with mental health problems will be assessed with an adapted version of the 10-item personal stigma subscale of the Generalised Anxiety Stigma Scale.^24^ In the adapted version of the scale, ‘anxiety disorder’ will be replaced with ‘mental health problem’ in each item. Items are responded to on a 5-point scale ranging from 0 (strongly disagree) to 4 (strongly agree). Responses to each of the items are summed to generate a total score ranging from 0 to 40, with higher scores indicating higher levels of mental health stigma. Internal consistency for this scale is high (Cronbach α=0.86).^24^

#### Suicide stigma

The stigma (eight items) and glorification and normalisation (four items) subscales from the Stigma of Suicide Scale-Short Form^25^ will be used to assess attitudes towards suicide. Participants rate their level of agreement with 1–2 word descriptors of a person who dies by suicide on a 5-point Likert scale ranging from 1 (strongly disagree) to 5 (strongly agree). Item scores for each subscale are summed to generate a total score ranging from 8 to 40 for the stigma subscale and 4–20 for the glorification and normalisation subscale. Higher scores on each subscale indicate higher levels of suicide stigma and the glorification and normalisation of suicide. The stigma (α=0.89) and glorification and normalisation (α=0.82) subscales have shown high internal consistency.^26^

#### Help-seeking attitudes

Help-seeking attitudes will be assessed using a modified version of the 10-item Attitude Towards Seeking Psychological Professional Help Scale-Short Form (ATSPPH-SF).^27 28^ Seven scale items have been adapted to elicit a parent’s attitudes towards help-seeking for their child (eg, ‘I would want to get psychological help if my child was worried or upset for a long period of time’). Each item is rated on a 4-point Likert scale ranging from 0 (disagree) to 3 (agree). Five items are reverse-scored prior to summing item scores to generate a total scale score ranging from 0 to 30. Higher scores indicate more positive attitudes towards seeking professional help. Previous research using the ATSPPH-SF has reported high internal consistency (α=0.84).^29^

#### Barriers to help-seeking

To assess parent perceptions of modifiable barriers to help-seeking, a list of 11 barriers was developed based on items from the Barriers to Access to Care Evaluation,^30^ and previous research investigating help-seeking barriers.^29 31^ Example items include ‘I would prefer to work it out as a family’ and ‘Seeking help might impact my child’s future prospects’. Each item is responded to on a 5-point scale ranging from 1 (not true for me) to 5 (true for me). Item scores are summed to form a total scale score ranging from 11 to 55, with higher scores indicating higher perceived barriers to help-seeking.

#### Help-seeking intentions

The General Help-Seeking Questionnaire^32^ will be used to measure help-seeking intentions for a child’s personal or emotional problems. Intentions to seek help from 13 different formal and informal sources (eg, intimate partner, friend, general practitioner and teacher) are assessed on a 7-point Likert scale ranging from 1 (extremely unlikely) to 7 (extremely likely). An additional item will also assess the likelihood of not seeking help from anyone.

#### Psychological distress

The Distress Questionnaire 5^33^ will be used to assess participants’ general psychological distress. The scale investigates psychological distress in the last 30 days, with items rated on a scale from 1 (never) to 5 (always). Item scores are summed to generate a total score ranging from 5 to 25, with higher scores reflecting greater psychological distress. Internal consistency for this scale is high (α=0.86).^33^

#### Resource satisfaction, acceptability and engagement

Among intervention condition participants, the level of engagement with the resources will be assessed at postintervention using resource backend data (eg, logins and completion), as well as self-report data on the number of times the resource was accessed, time spent on the resource, if participants downloaded any information sheets and whether they shared the resource content with anyone. At follow-up, participants will be asked if they accessed the resource over the previous 12-week period and, if so, which sections and, why.

Satisfaction and acceptability of the mental health and suicide gatekeeper resource will also be assessed at postintervention using a satisfaction measure adapted from a previous study.^34^ Open-ended questions at postintervention will also be used to identify what participants liked most and least about the resource, what they found to be most useful and important, the likelihood of recommending the resource to other parents and possible resource changes or additions. A list of seven potential barriers to accessing the resource will also be presented to participants, who will be asked to select any relevant barriers, with a free-text response available to nominate other barriers.^35^

Semistructured interviews of approximately 60 min will also be conducted with 15–20 intervention-condition participants to further explore motivations to use the resource, what parts of the resource were most and least helpful, if they had shared their learning with anyone and possible resource refinements and dissemination pathways. We believe this number will be sufficient to explore the perspectives of parents with a range of mental health experiences.

#### Demographic information

At the preintervention assessment, participants will be asked to provide their age, gender (female, male, non-binary and use different terms), language spoken at home (English only, English and another language and another language only), state or territory of residence, residential location (metropolitan, regional, rural or remote), employment status, level of education, relationship status, number of children, number of primary-school-aged versus secondary-school-aged children, previous qualifications or training in mental health and mental health history of the parent.

#### Child mental health

At preintervention and to assist with randomisation, participants will be asked whether a child in their care has experienced a mental health problem and, if yes, whether their child received treatment. At the preintervention and follow-up assessments, participants will be asked whether they have been concerned about their child’s mental health in the last 3 months. Depending on the response, participants may be asked several follow-up questions about whether and why they have, or have not, spoken to their child about this concern, whether they supported their child in seeking help, what motivated them to take (or not take) action and who they sought help from. Participants will also be asked whether they have used any resources to help with their child’s mental health in the last 3 months and to specify which resources they have used.

### Sample size and power calculations

The calculation of the required sample size was based on detecting a moderate postintervention effect size (d=0.4) for change in self-efficacy between the intervention and waitlist control conditions. Power was set at 0.9, α=0.05 (two-tailed) and a correlation of 0.5 was assumed between preintervention and endpoint scores. Accommodating potential attrition rates between 30% and 50%, the target sample size is 380 participants (190 per condition).

### Data analysis plan

#### Attrition and preintervention comparisons

Logistic regression analyses will be conducted to identify significant predictors of attrition at postintervention and 12-week follow-up. Univariate mixed effect models will be used to identify any differences between the intervention and waitlist control conditions at preintervention.

#### Intervention effects

Statistical analysis of continuous measures will be undertaken on an intent-to-treat basis, including all participants randomised regardless of intervention completion or withdrawal from the study. Mixed-model repeated measures analysis of variance will be used, with measurement occasion as a within-groups factor and condition as a between-groups factor. This approach yields unbiased estimates of intervention effects under the assumption that data is missing completely at random or at random and will allow the inclusion of participants with missing data without using biased techniques, such as the last observation carried forward.^36^ For any dichotomous outcomes, a comparable binary mixed modelling approach^37^ will be used. Cohen’s d effect sizes will be calculated for each time point. If efficacy is demonstrated, exploration of potential mediators and moderators of response, such as child’s age, child’s mental health, participant gender and intervention completion, will be explored separately using three-way interaction terms and stratified models.

#### Satisfaction and acceptability

Descriptive statistics will be used to summarise resource use, resource satisfaction and acceptability. Deidentified transcribed interview data will be qualitatively analysed using framework analysis, with a focus on examining user experience, preferences for content and potential barriers to access. Framework analysis was designed to address social policy research questions^38^ and has been used frequently in health research.^39^ It follows a systematic process of inductive coding, generating a table of findings across participants that is conducive to interpretation and input from multiple researchers.

### Ethics and Dissemination

The study will establish the efficacy of the gatekeeper resource and will inform future resource revisions and dissemination pathways. The ethical aspects of the study were approved by the Australian National University Human Research Ethics Committee (Protocol 2023/195). Prior to participating, the participant information sheet will be displayed, with participants providing informed digital consent to participate. All participants included in the trial will have access to information on crisis support numbers and mental health websites for use if they are feeling distressed. This information will be included on the information sheet provided to participants, as well as at the end of each survey.

All research data will be securely stored at the Australian National University for at least five years from the date of any publication arising from the research and will be accessible to the research team. At the end of the five-year period, all electronic survey and transcription data will be archived in a deidentified format (all reasonably identifying information removed) and may be shared with other researchers, with permission from the original research team.

Results of the current study will be communicated in aggregate form to key stakeholders, parents/carers, mental health practitioners, education providers, and the academic community through community forums, academic conferences and peer-reviewed publications.

### Trial status

Recruitment for the trial commenced on 21^st^ September 2023, with recruitment aiming to be complete by the end of May 2024.

## supplementary material

10.1136/bmjopen-2023-082963 online supplemental file 1
