# Supplementary material for: Randomised controlled trial of an online mental health and suicide gatekeeper resource for parents and caregivers: study protocol
Source: BMJ Open. 2024 Jul 17;14(7):e082963. doi: 10.1136/bmjopen-2023-082963 (PMC11256068; doi:10.1136/bmjopen-2023-082963)
Supplement: online supplemental file 1 [file bmjopen-14-7-s001.pdf]

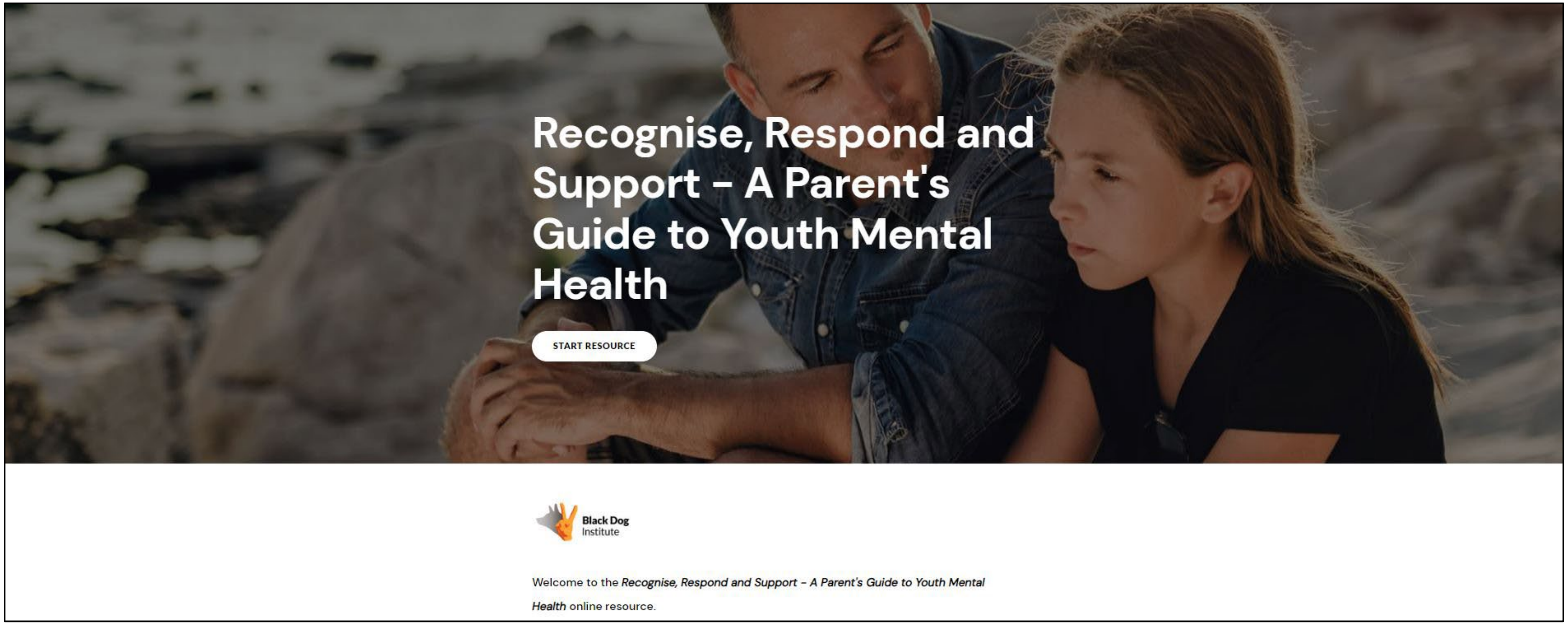

Figure 1: Cover page of Recognise, Respond and Support Resource (Image sourced from iStock).

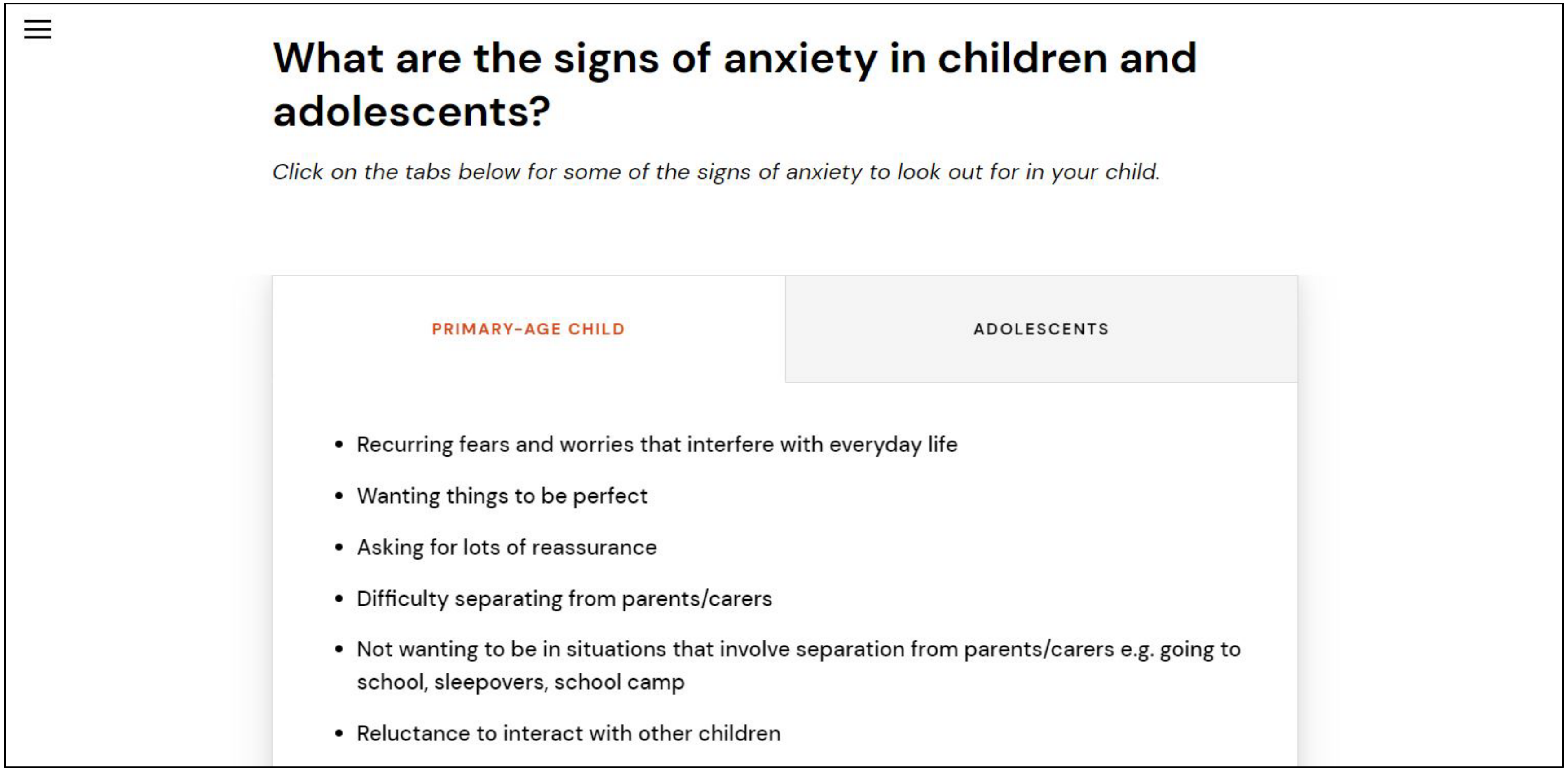

Figure 2: Tab structure to display signs of anxiety for children and adolescents.

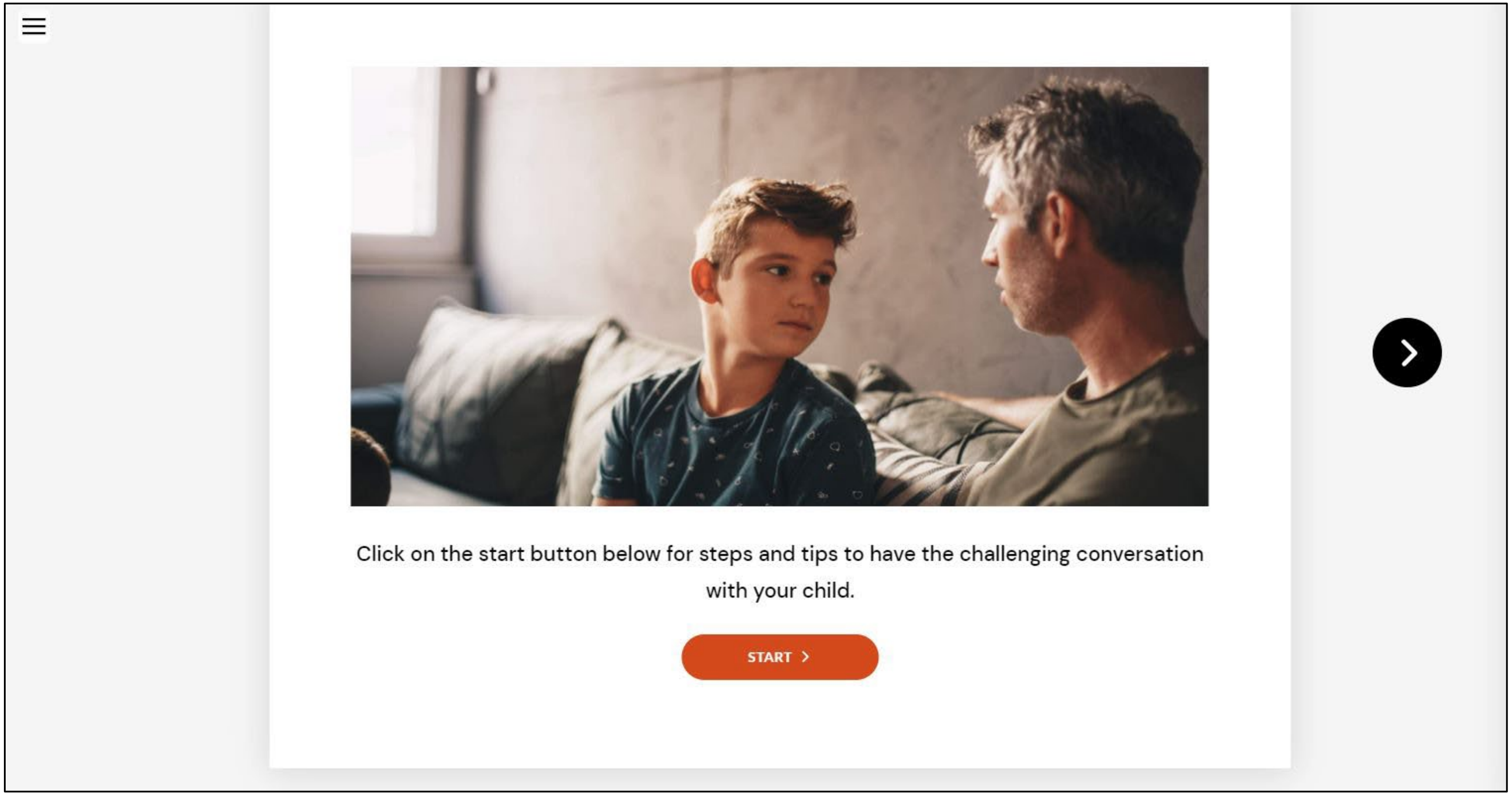

Figure 3: How to have a conversation with your child for the Respond component of the resource (Image sourced from iStock).

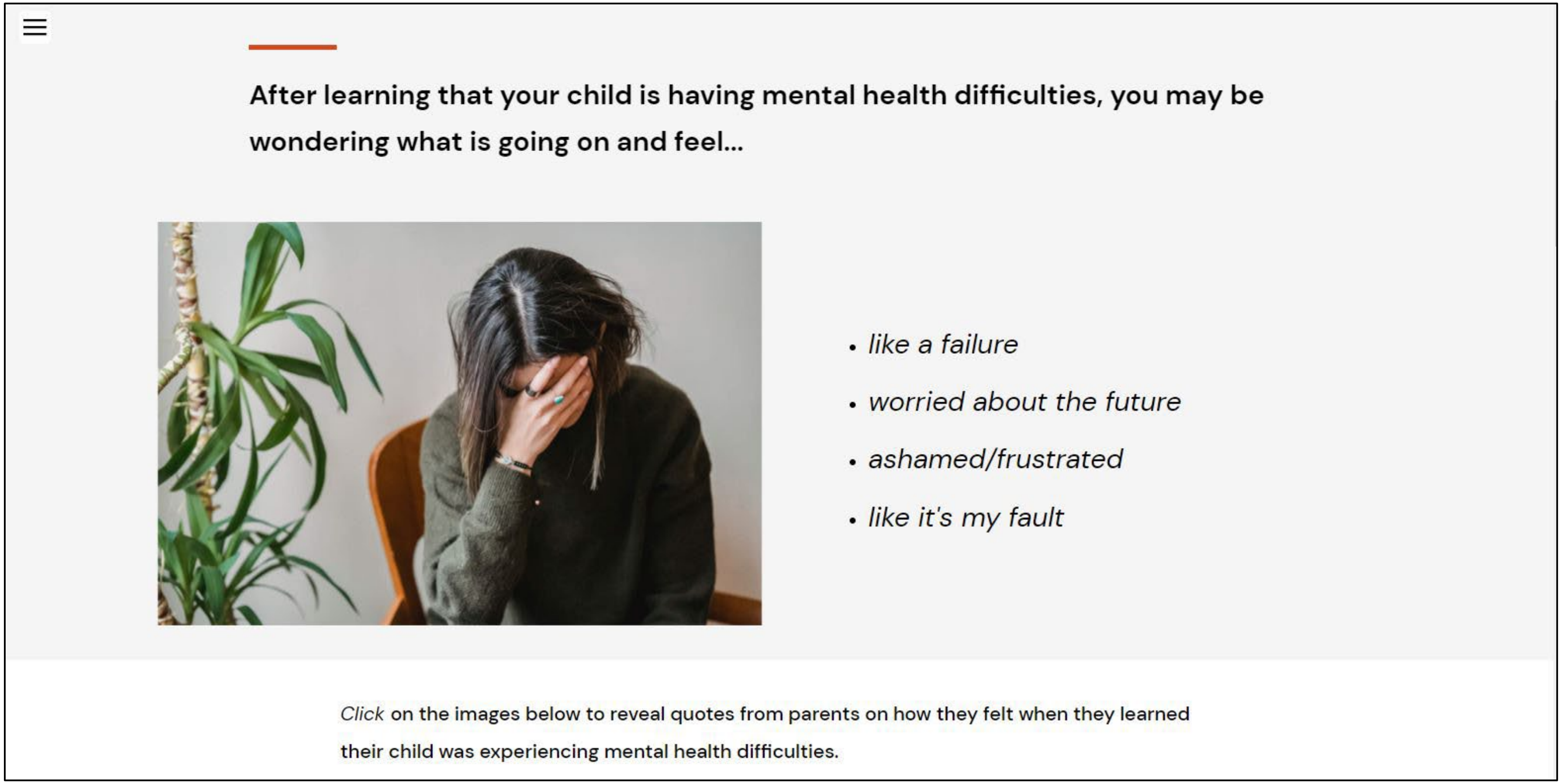

Figure 4: Emotions parents may feel when discovering their child is having mental health difficulties (Image sourced from iStock).

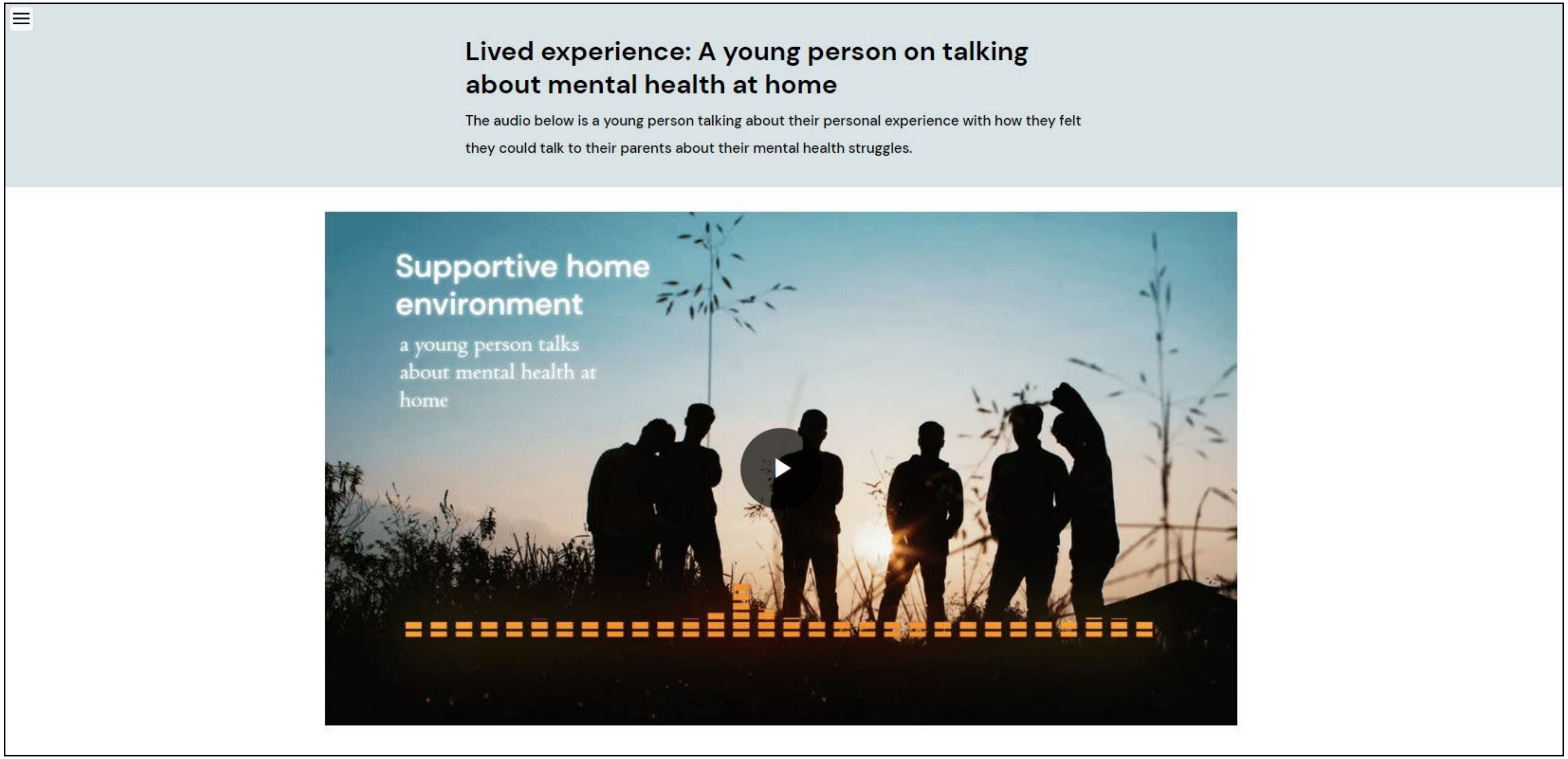

Figure 5: Audio files are included to provide lived experience (Image sourced from iStock).
