# Supplementary material for: Randomised controlled trial of an online mental health and suicide gatekeeper resource for parents and caregivers: study protocol
Source: BMJ Open. 2024 Jul 17;14(7):e082963. doi: 10.1136/bmjopen-2023-082963 (PMC11256068; doi:10.1136/bmjopen-2023-082963)
Supplement: Supplementary file 2 [file bmjopen-2023-082963.reviewer_comments.pdf]

PEER REVIEW HISTORY

BMJ Open publishes all reviews undertaken for accepted manuscripts. Reviewers are asked to complete a checklist review form (**http://bmjopen.bmj.com/site/about/resources/checklist.pdf**) and are provided with free text boxes to elaborate on their assessment. These free text comments are reproduced below.

ARTICLE DETAILS

**TITLE (PROVISIONAL)**	Randomised controlled trial of an online mental health and suicide gatekeeper resource for parents and caregivers: Study protocol
**AUTHORS**	Calear, Alison L.; McCallum, Sonia; Kazan, Dominique; Torok, Michelle; Werner-Seidler, Aliza; O'Dea, Bridianne; Morse, Alyssa; Farrer, Lou; Shand, Fiona; Batterham, Philip

VERSION 1 – REVIEW

**REVIEWER**	Villarreal-Otálora, Tatiana<br>Kennesaw State University
**REVIEW RETURNED**	28-Dec-2023

**GENERAL COMMENTS**	This is a well throughout and described protocol.

**REVIEWER**	Tuomainen, Helena<br>University of Warwick, Warwick Medical School
**REVIEW RETURNED**	29-Feb-2024

**GENERAL COMMENTS**	This is a very well written study protocol worthy of publication. The protocol describes an RCT of an intervention that is sorely needed as it provides an online resource to parents to help recognise, respond and access support for their child with mental health difficulties and/or suicidal thoughts. I have only a few comments/requests.<br><br>The developed intervention supports parents of children aged 5-17 years. This is a very wide age range. Further information is needed in the introduction about mental health problems of children at different ages (i.e. developmental psychopathology) and why parents might need a mental health and suicide gatekeeper resource for children as young as 5 years of age.<br><br>Additionally, the description of the intervention on p. 10 should explain whether and how the online resource takes the age of the child into account. The images provided are of older children.<br><br>It would also be interesting to know how this online intervention differs to the one reported on p. 6 L40, i.e. apart from age.<br><br>In the section on patient and public involvement (p. 11), further information about the diversity of the parents and caregivers who informed the development of the guide would be helpful. Is the guide appealing to all ethnic groups in Australia?

	I have not identified the date when recruitment was started. Rolling recruitment has been mentioned but an estimate of the length of recruitment would be helpful to know in the recruitment section.<br><br>The assessment battery is substantial and will need up to 30 minutes to complete. This may have a negative impact on retention at follow-up, but up to 50% attrition has been accounted for in the sample size calculation. The $15 gift card may not be a high enough incentive to motivate completion of the measures, but I hope I am wrong.<br><br>Will participants who take part in the qualitative interview get an incentive? If yes, this should be mentioned.<br><br>I wish the research team good luck with their trial and will await for the findings.

VERSION 1 – AUTHOR RESPONSE

Reviewer: 1
Dr. Tatiana Villarreal-Otálora, Kennesaw State University
Comments to the Author:
This is a well throughout and described protocol.
Thank you for reading our manuscript and for your positive comment.

Reviewer: 2
Dr. Helena Tuomainen, University of Warwick
Comments to the Author:
This is a very well written study protocol worthy of publication. The protocol describes an RCT of an intervention that is sorely needed as it provides an online resource to parents to help recognise, respond and access support for their child with mental health difficulties and/or suicidal thoughts. I have only a few comments/requests.
Thank you for your positive comments.

The developed intervention supports parents of children aged 5-17 years. This is a very wide age range. Further information is needed in the introduction about mental health problems of children at different ages (i.e. developmental psychopathology) and why parents might need a mental health and suicide gatekeeper resource for children as young as 5 years of age.
Thank you for this suggestion. We have added further information in the introduction to clarify the issues raised, including the dual focus of the resource on prevention and early intervention, as well as responding and supporting young people with existing mental health problems. The resource aims to build parental knowledge and supportive attitudes early on, as well as equip them with the skills to provide support when it is needed. As such, for a parent of a 5-year-old child, the content on suicide may not be relevant to them now, but it may serve to be helpful in the future when their child is older. It may also assist them to support other young people in their lives.

The introduction (page 6-7) has been revised to read: “In order to address these limitations, a new online gatekeeper intervention was developed in collaboration with end users, which specifically targets parents and caregivers and addresses common mental health problems in children and adolescents, as well as suicide and self-harm. To ensure its relevance to a wider range of parents/caregivers, the intervention was also designed to have a dual focus on upskilling parents/caregivers prior to difficulties arising through the building of knowledge and supportive

attitudes, as well as teaching skills to respond and support a young person with existing mental health problems."

Additionally, the description of the intervention on p. 10 should explain whether and how the online resource takes the age of the child into account. The images provided are of older children.
We have selected different screen captures of the resource now in the supplementary material section to reflect the resource more accurately. "Recognise" is the only section of the resource that has age specific information, the remainder of the resource has been designed for parents with children of all ages.

In the Recognise section parents can click a tab for information about signs of anxiety or depression in children and adolescents. To clarify this in the manuscript, the text has been changed slightly for the Recognise section (page 11) and now reads "Specific information is then provided on anxiety, depression, self-harm, and suicide and how to identify when help may be needed. Separate signs of anxiety and depression are listed for children and adolescents to provide age specific information for parents." For the supplementary materials, we have included a screenshot of the tabs used to display information for primary aged children and adolescents.

It would also be interesting to know how this online intervention differs to the one reported on p. 6 L40, i.e. apart from age.
The resource evaluated in the trial reported on page 6 is LivingWorks Start, which is specifically focused on increasing awareness of suicide and learning skills to communicate with someone considering suicide and connect them with help. Unlike our resource, it only focuses on suicide and not mental health more broadly. Further it is a generic community training program for adults and not specifically designed to support parents. We have now revised the text to make the differences between the interventions clearer (page 6) "Recently, a pre-post study was conducted to assess the efficacy of a generic online suicide prevention training program in a sample of parents of young people aged 12-25 years (16). The results of this study showed increases in participant self-efficacy and formal help-seeking intentions, and reductions in suicide stigma at post-intervention, and increases in suicide literacy at 3-month follow-up. While the results of this study are promising, the constrained focus on suicide, the use of a program not explicitly tailored for parents, and the pre-post design of the study are inherent limitations."

In the section on patient and public involvement (p. 11), further information about the diversity of the parents and caregivers who informed the development of the guide would be helpful. Is the guide appealing to all ethnic groups in Australia?
We obtained a diverse sample of participants and caregivers in the survey used to guide the development of the resource (based on their language spoken at home, location of residence, education level, mental health history). The Patient and Public Involvement section on page 12 has been revised to explain this and now reads "This led to recruitment of a diverse sample of parents and caregivers through social media to engage in the completion of a survey (n = 631) to identify the target disorders (anxiety, depression, self-harm and suicide) and population (both children and adolescents) of the resource, as well as its format (digital self-help), key informational needs in the areas of recognition, response and support, and an assessment of potential target areas (literacy and stigma) to identify areas for improvement."
Once the effectiveness of the base program has been established, further work with specific populations e.g., disadvantaged, ethnic groups could be undertaken to meet the needs of these populations.

I have not identified the date when recruitment was started. Rolling recruitment has been mentioned but an estimate of the length of recruitment would be helpful to know in the recruitment section.

This has been added under the section ‘Trial Status’ in response to a comment from the editor.

The assessment battery is substantial and will need up to 30 minutes to complete. This may have a negative impact on retention at follow-up, but up to 50% attrition has been accounted for in the sample size calculation. The $15 gift card may not be a high enough incentive to motivate completion of the measures, but I hope I am wrong.
Thank you for your helpful feedback. Our estimates were based on experience from previous trials that were conducted online, as well as ethical considerations in reducing risks of coercion.

Will participants who take part in the qualitative interview get an incentive? If yes, this should be mentioned.
Yes, participants in the qualitative component will be offered a $40 e-gift card. This has now been mentioned in the last paragraph of the Procedure section (page 10) and reads “Participants will be offered a $40 e-gift card to compensate them for their time.”

I wish the research team good luck with their trial and will await for the findings.
Thank you
